# Current Insights and Future Directions in the Treatment of Heart Failure with Preserved Ejection Fraction

**DOI:** 10.3390/ijms25010440

**Published:** 2023-12-28

**Authors:** Roxana Mihaela Chiorescu, Roxana-Daiana Lazar, Alexandru Ruda, Andreea Paula Buda, Stefan Chiorescu, Mihaela Mocan, Dan Blendea

**Affiliations:** 1 Department of Internal Medicine, “Iuliu Hațieganu” University of Medicine and Pharmacy, 400012 Cluj-Napoca, Romania; roxana.chiorescu11@gmail.com; 2 Department of Internal Medicine, Emergency Clinical County Hospital, 400006 Cluj-Napoca, Romania; 3 Nicolae Stăncioiu Heart Institute, 400001 Cluj-Napoca, Romania; alexandruruda@yahoo.com (A.R.); buda_andreeapaula@yahoo.com (A.P.B.); dblendea1@gmail.com (D.B.); 4 Department of Surgery, “Iuliu Hațieganu” University of Medicine and Pharmacy, 400139 Cluj-Napoca, Romania; stefanc74@yahoo.com; 5 Department of Cardiology, “Iuliu Hațieganu” University of Medicine and Pharmacy, 400437 Cluj-Napoca, Romania

**Keywords:** heart failure, ejection fraction, microARN, serpina, melatonina

## Abstract

Heart failure is a clinical syndrome associated with poor quality of life, substantial healthcare resource utilization, and premature mortality, in large part related to high rates of hospitalizations. The clinical manifestations of heart failure are similar regardless of the ejection fraction. Unlike heart failure with reduced ejection fraction, there are few therapeutic options for treating heart failure with preserved ejection fraction. Molecular therapies that have shown reduced mortality and morbidity in heart failure with reduced ejection have not been proven to be effective for patients with heart failure and preserved ejection fraction. The study of pathophysiological processes involved in the production of heart failure with preserved ejection fraction is the basis for identifying new therapeutic means. In this narrative review, we intend to synthesize the existing therapeutic means, but also those under research (metabolic and microRNA therapy) for the treatment of heart failure with preserved ejection fraction.

## 1. Introduction

Heart failure (HF) is a clinical syndrome (symptoms: breathlessness, fatigue, and ankle swelling; and signs: peripheral edema, pulmonary crackles, and elevated jugular venous pressure) due to a structural and/or functional abnormality of the heart that results in a symptomatic increase in heart-filling pressures and/or inadequate cardiac output at rest and/or during exercise [1].

Depending on the ejection fraction (EF) of the left ventricle (LV), HF is classified into HF with preserved EF (HFpEF) over the cut-off limit of 50%, HF with slightly reduced EF (HFmrEF), between 40–50%, and HF with reduced EF (HFrEF) less than 40% [1].

Although both clinical manifestations and hospitalization rates of HFpEF are very similar to HFrEF, the therapeutic response to molecular therapies differs immensely. According to RCT like CHARM-PRESERVED [2], I-PRESERVE [3], PEP-CHF [4], and TOPCAT [5], therapies with proven effects on reducing morbi-mortality in HFrEF, such as angiotensin-converting enzyme inhibitors, beta-adrenergic blockers, angiotensin II receptor blockers have not shown similar benefit in HFpEF. It is worth mentioning that in these RCTs, the cut-off for HFpEF varied between 40%, 45%, and 50%.

Though disabled by symptoms and with an increased risk of mortality, patients diagnosed with HFpEF have few therapeutic options. Recent guidelines (ESC 2023) recommendations are focusing on the use of class I drugs for HFpEF: diuretics (including mineralocorticoid antagonists), Sodium-Glucose Transport Protein 2 (SGLT2)inhibitors together with specific drugs for cardiac and noncardiac comorbidities [6,7].

Research into the pathophysiological mechanisms involved in HFpEF represents the basis for identifying new therapeutic methods. In the research race are metabolic and microRNA therapy. These therapies require validation through clinical trials to be introduced into current practice.

The present review aims to summarize the main therapeutic means existing for HFpFE, but also those under research, presenting the physiopathogenetic mechanisms underlying their use.

## 2. Main Pathophysiological Mechanisms Involved in the Production of HFpEF

Unlike HFrEF, in HFpEF the contractile function is preserved, but compliance is low due to ventricular myocardial remodeling that causes large increases in filling pressure with minimal dilation and preserved flow rate. Thus, retrograde stasis occurs in a cavity slightly or not dilated at all [8].

In HFrEF, there is an impairment of the overall contractility of a ventricle, with a relatively small decrease in diastolic ventricular compliance. The compensatory diastolic mechanism is in great demand, cavitary dilation is high, and only after overcoming this mechanism does the increase in filling pressure and decrease in flow become apparent [9].

### 2.1. Ventricular Remodeling

The following processes underlie the production of myocardial remodeling and cardiomyocyte dysfunction in HFpEF:

1. A systemic proinflammatory state associated with comorbidities (e.g., obesity, arterial hypertension, diabetes mellitus, etc.) [10,11,12];
2. As a consequence of the systemic proinflammatory state, oxidative stress is primed in endothelial cells, and reactive oxygen species (ROS) are produced, limiting the bioavailability of nitric oxide (NO) from adjacent cardiomyocytes [10];
3. Decreased bioavailability of NO in cardiomyocytes decreases protein kinase G (PKG) activity;
4. Decreased activity of PKG in cardiomyocytes causes hypophosphorylation of cytoskeletal protein-titin, thereby inducing concentric remodeling of LV and hardening of cardiomyocytes;
5. Both rigid cardiomyocytes and secondary increased collagen deposition by myofibroblasts cause diastolic dysfunction LV, the primary cardiac functional deficit in HFpEF [10].

**Figure 1:**
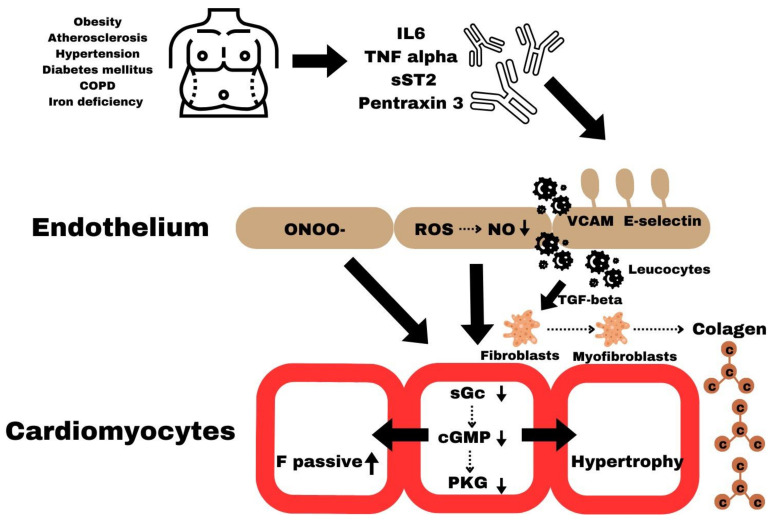
The main pathophysiological process involved in the development of HFpEF [10].

Myocardial remodeling in HFpEF differs from myocardial remodeling in HFrEF, which is caused by the death of cardiomyocytes due to oxidative stress caused by various factors such as ischemia, infection, and toxicity (Figure 1) [13,14,15].

### 2.2. Retrograde Stasis

The alteration of diastolic function as a result of cardiac remodeling causes increased preload, i.e., filling pressure in the respective cardiac cavity, with the retrograde transmission of pressure in the respective venous system (systemic or pulmonary), passive dilation of veins, and stasis. Thus, the clinical symptoms and signs of right or left HF appear. Overall, diastolic HF means increased filling pressure in both systems [10,13].

### 2.3. Pulmonary Hypertension

Pulmonary hypertension (PH) can occur in patients with HFpEF through several mechanisms: increased pressure in the left atrium, pulmonary vasoconstriction, and pulmonary vascular remodeling. As a result of PH, right ventricular dysfunction occurs, which is associated with a significantly increased risk of mortality [10].

### 2.4. Coronary Microvascular Dysfunction

Coronary microvascular dysfunction refers to abnormalities in the small blood vessels of the heart, particularly those with diameters less than 500 μm. In the context of HFpEF, this dysfunction is increasingly recognized as a significant contributor to the pathophysiology [16]. Coronary microvascular dysfunction in HFpEF is often associated with impaired coronary flow reserve, meaning that the coronary circulation has difficulty adjusting blood flow in response to increased demand, such as during exercise [17]. The impaired microvascular function can lead to suboptimal perfusion of the myocardium, resulting in episodes of myocardial ischemia [18]. Over time, this ischemia-reperfusion injury contributes to myocardial fibrosis, a common feature in HFpEF [18]. Inflammation plays a role in both coronary microvascular dysfunction and HFpEF [19]. Inflammatory processes can contribute to endothelial dysfunction and oxidative stress, further compromising the function of the microvasculature [18]. Dysfunction of the endothelium, the inner lining of blood vessels, is a central feature of microvascular dysfunction. In HFpEF, endothelial dysfunction contributes to impaired vasodilation and abnormal regulation of blood flow, as noted above [13].

### 2.5. Chronotropic Incompetence

In a healthy cardiovascular system, the heart rate increases appropriately in response to physical activity. This adaptive response ensures sufficient blood supply to meet the increased metabolic demands of the body. However, in chronotropic incompetence, this response is blunted or delayed, leading to a mismatch between the heart’s pumping capacity and the body’s requirements. The molecular mechanism underlying this process is impaired mitochondrial function, which contributes to energy depletion in cardiomyocytes [20]. This deficiency further compromises the heart’s ability to meet the increased demands, especially during exercise. This limitation in filling, combined with chronotropic incompetence, creates a double burden on the heart during periods of increased demand, such as exercise. Chronotropic incompetence not only compromises the heart’s ability to respond to stress but also exacerbates the limitations imposed by diastolic dysfunction [13].

### 2.6. Skeletal Muscle Damage

Skeletal muscle mass is decreased, fatty infiltration increases, and capillary density decreases in patients with HFpEF. Oxidative metabolism of skeletal muscle fiber is reduced by alteration of mitochondrial number and function [20]. These changes cause a decrease in the exercise capacity of these patients [13]. Bowen et al. tested an animal model to elucidate the “potential molecular, mitochondrial, histological, and functional alterations induced by HFpEF in the diaphragm and soleus” of female rats [20]. The findings highlight the association between a high-salt diet, HFpEF-like phenotype, and mitochondrial dysfunction, particularly in the diaphragm. Exercise training appears to have a beneficial effect on mitochondrial function in this rat model, emphasizing the potential role of physical activity in mitigating HFpEF-related changes [21]. These studies have valuable insights into the molecular mechanisms associated with mitochondrial dysfunction in HFpEF, emphasizing the importance of exploring therapeutic strategies, such as exercise training, in addressing these abnormalities.

## 3. Main Phenotypes in HFpEF

**Figure 2:**
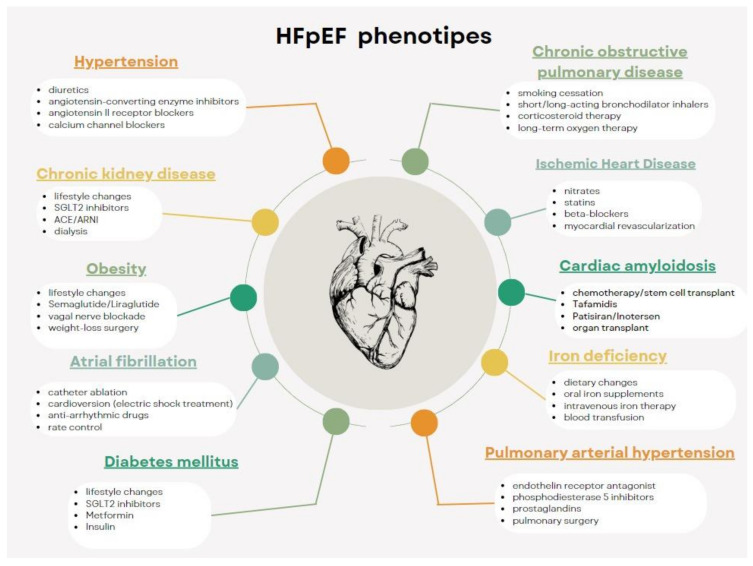
The main phenotypes and suitable treatment in patients with HFpEF [22].

Depending on the associated comorbidities, patients with HFpEF have several phenotypes, as illustrated in Figure 2. Thus, we included patients with HFpEF and hypertension, renal dysfunction, diabetes, obesity, chronic obstructive pulmonary disease, obstructive sleep apnea, ischemic coronary artery disease, iron deficiency, cardiac amyloidosis, pulmonary arterial hypertension, cachexia, chronotropic incompetence, and/or older age (>65 years) [22].

Several classifications of HFpEF that are useful in establishing therapeutic strategies and developing new therapies are described in the literature.

From a pathophysiological point of view, the following subtypes of HFpEF are identified:

**A subtype of Left Atrial Myopathy**

In the phenotype of left atrial myopathy as a result of the action of cardiovascular risk factors and especially hypertension, patients develop left atrial dilation, mitral insufficiency, and atrial fibrillation. The LV’s preserved systolic and diastolic function is detected but with a more pronounced impairment of cardio-vascular hemodynamics: increased pulmonary arterial pressure and low VS beat volume. This phenotype is associated with increased natriuretic peptides and other circulating proteins such as angiotensin convertase enzyme 2 (ACE2). As drug therapy, macitentan (an antagonist of endothelin A and B receptors) is effective. Interatrial shunt devices and procedures have been studied for patients who fit this phenotype.

**Genetic subtype**

This subtype includes patients with left ventricular hypercontractility or hypo-contractility despite preserved FE. These patients, and predominantly male patients, do not respond favorably to conventional treatment for HF. Myosin inhibitors represent a proposed therapeutic condition for this subtype of patients.

**Molecular subtype: plasminogen activator-1 inhibitor**

PAI-1 is a protein secreted by visceral adipose tissue found in most patients with HFpEF. Clinical studies have demonstrated its role as a promoter of senescence. Patients included in this subtype are characterized by metabolic comorbidities, systemic inflammation, and accelerated aging.

Identifying and antagonizing pathophysiological pathways through which PAI-1 acts represents new therapeutic directions. Cardiovascular risk factors/patient comorbidities cause increased inflammation and oxidative stress at the cellular level and increased production of PAI-1 and other related proteins by visceral adipose tissue. A rare variant of the gene coding for PAI-1, SERPINE 1, is associated with PAI-1 deficiency.

Another etiopathogenetic classification divides patients with HFpEF into five subtypes:

Phenotype 1. Morbid obesity and natriuretic peptide deficiency syndrome. It includes patients with obesity, insulin resistance, and hyperaldosteronism. Weighted decrease by decreased caloric intake, increased physical activity, and the use of GLP-1 receptor agonists have beneficial effects in these patients.

Phenotype 2. Cardio-metabolic syndrome (includes patients with diabetes, hypertension, and obesity). The pathophysiological mechanism involved consists of excessive glycation of proteins that cause dysfunction in the microvasculature and peripheral vessels, myocardial stiffness, and increased interstitial fibrosis. So far, studies have shown that fighting cardiovascular risk factors alone is not enough to combat the cardiac and systemic changes that these cardiovascular risk factors have produced.

Phenotype 3. Renal failure. It is characterized by endothelial dysfunction, decreased cardiac output, and increased central venous pressure.

Phenotype 4. Previously discussed left atrial myopathy (includes hypertensive patients with atrial fibrillation and left atrial dilation).

Phenotype 5. PH. They are pathophysiologically characterized by endothelial dysfunction, pre- or post-capillary PH, and right ventricular dilatation/insufficiency. Soluble guanylcyclase stimulators have not demonstrated their effectiveness in Vitality-HFpEF studies and Socrate Preserved [22,23].

## 4. Diagnosis of HFpEF

**Figure 3:**
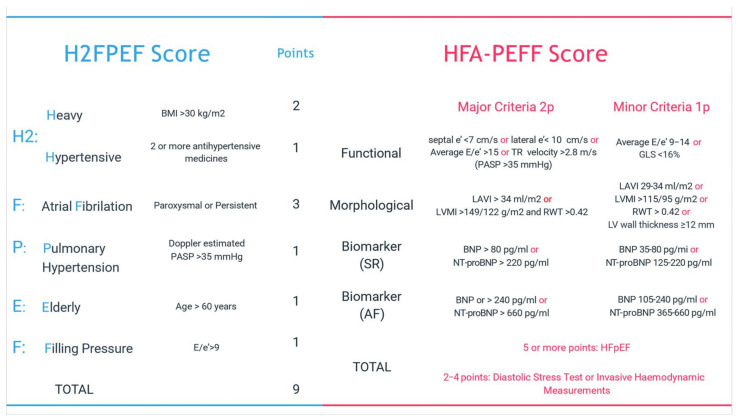
The scores used to estimate the probability of underlying HFpEF [6,7].

1. It involves identifying characteristic symptoms of HF: effort dyspnea, orthopnea, etc. Pathologies with similar signs and symptoms will be excluded (renal/hepatic failure, chronic obstructive pulmonary disease, venous insufficiency [6]);
2. In addition to the presence of characteristic signs and symptoms, for the diagnosis of HFpEF the following elements are necessary: a normal systolic function and evidence of left ventricular (LV) diastolic dysfunction or raised LV filling pressures (e’septal < 7 cm/s, e’lat < 10 cm/s, average E/e’ > 14, LA volume index > 34 mL/mp, peak TR velocity > 2.8 m/s) [24];
3. Elevated serum levels of natriuretic peptides support; however, normal levels do not rule out a diagnosis of HFpEF as natriuretic peptides are largely influenced by the presence of obesity, gender, age, and kidney function [9];
4. Non-invasive diagnosis or exclusion of HFpEF will not depend on a single parameter above or below a certain threshold but on a combination of parameters derived from clinical, laboratory, and imaging tests that together will give a probability for diagnosis. For normal LV filling pressure or a non-conclusive evaluation to estimate the probability of underlying HF, there are scores like H2FPEF score (recommended by the American Society of Cardiology) and HFA-PEFF score (recommended by the European Society of Cardiology) (Figure 3) or diastolic stress test [24].
5. Other entities with preserved EF that should also be excluded from the diagnosis of primary HFpEF are as follows: valvular disease, congenital disease, constrictive pericarditis, restrictive cardiomyopathy, hypertrophic cardiomyopathy, storage disease, ischemic heart disease, high output HF, and primary right ventricular failure with similar symptoms [25].
6. HPpEF phenotypes for targeted therapy should be identified (Figure 2) [22].

We mention that current management guidelines recommend that for the diagnosis of primary HFpEF, we exclude patients with pathologies characterized by diastolic dysfunction determined by specific etiologies: cardiac amyloidosis, hypertrophic cardiomyopathy, pericardial diseases, valvular diseases, etc. This recommendation is based on the fact that these entities present particular means of treatment and prognosis [22]. In contrast, older studies looking at the effectiveness of renin-angiotensin-aldosterone inhibitors did not exclude these categories of cardiovascular disease with poor prognosis because the initial concept of the pathophysiological mechanism of HFpEF considered primary cardiac injury manifested by ventricular hypertrophy and diastolic dysfunction as the primary cause of HFpEF syndrome [23].

## 5. Treatment of Patients with HFpEF

Clinical studies that included HFpF patients have not demonstrated the mortality- and morbidity-lowering benefits **of ACE inhibitors or sartans**, compared to patients with HFrEF, to which these classes of drugs have well-known benefits. The Charm-Preserved trial compared the effects of candesartan with placebo in patients with HF and LVEF >40%. There was only a small reduction in hospitalization rates and overall morbidity and no reduction in overall mortality [2]. The PEF-CHF study compared the effects of perindopril with placebo in HF patients older than 70 years with LVEF >45%, but there was no reduction in mortality and cardiovascular hospitalizations in all PFH patients [4]. Irbesartan also failed to reduce primary endpoints (cardiovascular mortality and hospitalization) in HFpEF patients compared to placebo in the Preserve trial [3].

The Treatment of HFpEF with a mineralocorticoid receptor antagonist (MRA) was evaluated extensively by TOPCAT trial which investigated the effect of **spironolactone** compared to placebo in patients with HFpEF. After a follow-up period of 3.3 years, there was no benefit on mortality and morbidity between the two groups, although hospitalization rates in the spironolactone group were less frequent than placebo. The spironolactone group had double rates of hyperkalemia and higher serum creatinine levels. These results suggest that spironolactone could be used in selected groups of patients whose creatinine and potassium levels can be closely monitored. MRAs also reduce serum markers of collagen synthesis in patients with cardiovascular disease, including HFrEF and HFpEF, which could reflect favorable effects on fibrosis [5].

The role of **beta blockers** in HFpEF is not well established. They may be beneficial by increasing ventricular filling time, reducing myocardial oxygen consumption, and controlling blood pressure [26,27].

In HFrEF, decreased cardiac output causes activation of the sympathetic nervous system, the renin-angiotensin-aldosterone system, the arginine-vasopressin system, natriuretic peptides, and endothelin, all of which cause water and salt retention in the body. Thus, activation of the renin-angiotensin-aldosterone system is a primary mechanism in HFrEF, and inhibition of its overexpression is a primary means of treatment in this category of patients [15].

HFpEF includes a greater variety of pathophysiological mechanisms with different specific treatments and a higher prevalence of non-cardiac comorbidities with systemic effects.

Unlike HFrEF, where the myocardial injury occurs secondary to direct cardiomyocyte injury (e.g., myocardial ischemia), in HFpEF, myocardial injury is not the primary source of disease. Still, it is the result of the cumulative effects of different comorbidities on the cardiovascular system [23].

Epidemiologically, comorbidities associated with patients with HFpEF have changed over time, with uncontrolled hypertension or smoking initially predominating as the main factors for HFpEF. These factors, over time, have been primarily controlled. Nowadays, common comorbidities are obesity, diabetes, atrial fibrillation, and the advanced age of the population. These comorbidities have been associated more frequently with HFpEF patients than with HFrEF patients in studies, and these comorbidities influence, to a greater extent, the unfavorable prognosis of these patients [23].

It should also be noted that clinical trials in patients with HFpEF show significant variability in response to treatment because patient selection was heterogeneous over time, depending on the type of clinical trial and the primary endpoints of different studies. There were differences in the EF of patients included in the study (40–50%) plus phenotypic variability of the patient [23].

Even for **Ca blockers in the** Optimaze—HF study, a reduction in mortality and a reduction in hospitalizations was not achieved [28].

One study showed that sildenafil (**phosphodiesterase inhibitor—PDE-5**), improved diastolic function and ***effort*** tolerance in patients with HFpEF and associated PH. A trial looking at the introduction of sildenafil in HFpEF patients without PH did not show a favorable outcome in terms of improving exercise capacity [29].

Use **of the combination of neprilysin**, **an sartan, and MRA** should be considered across the entire HFrEF. Among patients with HFpEF, women have a better response to these therapies. Women tend to have smaller LV chamber sizes (an LVEF at 50–55% in a woman may be abnormally low compared with a man) and a potentially different response to therapies with effects on the neurohormonal system [6].An If channel inhibitor with bradycardic effects improved exercise capacity in HFpEp patients [1].

**Diuretic drugs** are an essential symptomatic treatment in the presence of pulmonary or systemic stasis. Two classes of diuretics are mainly used: loop diuretics and thiazide diuretics. The lowest dose that prevents hydro-saline retention should be used, as overly high doses may lead to hydro-electrolyte imbalances (hyponatremia, hypokalaemia) and decreased organ perfusion (acute renal failure). If the glomerular filtration rate is lower than 30 mL/min, thiazides are contraindicated, and loop diuretics should be used [7].

In more advanced forms, insufficient response to diuretic treatment may occur, and resistance to diuretic treatment may occur. In this situation, it is necessary to check compliance with treatment and a low-sodium diet. In the case of right HF, intestinal edema may be responsible for the inadequate absorption of diuretics. In this case, an intravenous diuretic or a diuretic with increased oral bioavailability should be used. Another cause of diuretic resistance may be reduced renal perfusion, which leads to inadequate secretion of the diuretic. Doubling the dose of the diuretic is more appropriate than taking the same dose multiple times. The combination of a thiazide diuretic with a loop diuretic will potentiate the action of the loop diuretic [10].

Monitoring of diuretic treatment requires dosing serum levels of potassium, creatinine, urea, and uric acid. It is important to avoid volume depletion, which can lead to hypotension and renal dysfunction. Patients with hepatorenal syndrome should avoid the administration of nephrotoxic drugs [6,7].

**SGLT2 inhibitors**, originally developed for the treatment of diabetes, have garnered attention for their cardiovascular benefits beyond glycemic control. Recent studies have provided compelling evidence supporting their efficacy in HFpEF, a condition for which treatment options have historically been limited.

One notable study is the EMPEROR-Preserved trial, which investigated the SGLT2 inhibitor empagliflozin in patients with HFpEF. The trial demonstrated a significant reduction in the composite endpoint of cardiovascular death or hospitalization for HF in patients receiving empagliflozin compared to those on a placebo. This landmark trial highlighted the potential of SGLT2 inhibitors in improving outcomes in HFpEF [30].

Another significant trial is the DELIVER trial, which focused on dapagliflozin. In this study, dapagliflozin showed promising results in reducing symptoms and improving exercise capacity in patients with HFpEF. These findings contribute to the growing body of evidence supporting the use of SGLT2 inhibitors as a therapeutic option for HFpEF patients. The mechanisms through which SGLT2 inhibitors exert their beneficial effects in HFpEF are crucial for researchers to understand the underlying principles [31,32].

SGLT2 inhibitors induce glycosuria, leading to osmotic diuresis and natriuresis. This effect helps alleviate volume overload, a common feature in HFpEF. These inhibitors have been shown to improve ventricular hemodynamics by reducing preload and afterload, thereby enhancing cardiac efficiency [32].

Emerging evidence suggests that SGLT2 inhibitors may have anti-fibrotic and anti-inflammatory properties, which could contribute to mitigating the underlying structural changes associated with HFpEF. Beyond their direct cardiac effects, SGLT2 inhibitors modulate metabolism, reducing adiposity and improving insulin sensitivity, potentially addressing metabolic abnormalities often present in HFpEF [32].

As researchers, it’s essential to consider these mechanisms when exploring the potential use of SGLT2 inhibitors in HFpEF. The positive outcomes observed in recent trials underscore the need for further investigation and support the integration of these agents into the management of HFpEF.

One of the possible reasons why drugs used in clinical trials have not been shown to reduce morbidity-mortality of patients with HFpEF would be the heterogeneity of this entity in which pathologists with different pathologies are included. Among the causes of HFpEF are hypertension, obesity, diabetes, and myocardial ischemia [22].

Thus, phenotypic characterization of HF subtypes is very important. Regardless of phenotype, diuretics are used to reduce filling pressures. It is important to maintain sinus rhythm given the contribution of atrial systole to ventricular filling, and in the case of atrial fibrillation, it is important to control the ventricular frequency [6].

**GLP-1 RA** (Glucagon-like peptide 1 receptor agonists), initially developed for the management of diabetes [32] and for obesity treatment, have also shown promise in cardiovascular outcomes, including HF. Recent studies have shed light on their potential benefits in HFpEF, providing new avenues for therapeutic intervention.

The Functional Impact of GLP-1 RA for Heart Failure Treatment (FIGHT) trial is one example worth noting. This trial investigated the effects of the GLP-1 RA liraglutide in patients with HFpEF. Results indicated improvements in exercise capacity and quality of life in the liraglutide-treated group compared to placebo, suggesting a potential role for GLP-1 RAs in addressing the functional aspects of HFpEF [32].

The GALILEO trial is another relevant study in this context. While not exclusive to HFpEF, it included a subgroup analysis of HFpEF patients. The trial assessed the GLP-1 RA albiglutide and found a reduction in HF hospitalizations in the treatment group compared to placebo, emphasizing the cardiovascular benefits of GLP-1 RAs. Now, let’s explore some of the mechanisms through which GLP-1 RAs may exert their positive effects in HFpEF:

GLP-1 RAs have been shown to have direct inotropic and lusitropic effects on the heart, improving both contraction and relaxation. In HFpEF, where diastolic dysfunction is a prominent feature, these effects can contribute to enhanced cardiac function [32].

GLP-1 RAs exhibit metabolic benefits, including glucose-lowering and potential anti-inflammatory effects. These properties may be particularly relevant in the context of HFpEF, where metabolic abnormalities and inflammation are often observed [32].

GLP-1 RAs have been associated with improvements in endothelial function, which can have positive implications for vascular health in HFpEF patients [32].

As researchers, it’s crucial to consider these mechanisms and the clinical evidence when exploring the role of GLP-1 RAs in HFpEF therapy. While the field is still evolving, the findings from trials like FIGHT and GALILEO offer promising insights into the potential benefits of GLP-1 RAs for HFpEF patients [32].

Microvascular dysfunction is a predominant pathogenetic mechanism in HFpEF associated with myocardial fibrosis. It is an important prognostic factor for patients with HFpEF, correlating with the occurrence of adverse cardiovascular events such as hospitalization or cardiac death. It is also a possible treatment target. Among the possible therapeutic means of diastolic dysfunction, ACE inhibitors, sartans and beta-blockers have shown limited efficacy. Beneficial effects have been shown in SGLT2 inhibitors therapy, and there is hope for antifibrotic agents used for pulmonary fibrosis [33].

In conclusion, the treatment recommended by the guidelines for patients with HFpEF are as follows:

1. SGLT2 inhibitors is recommended as the first line of treatment in patients with HFpEF. Subsequently, diuretics will be added to patients with signs of congestion [7].
2. The main comorbidities will be identified, and the treatment will be adapted according to them (Figure 2).
3. In patients who remain symptomatic, MRA and/or RNAs should be considered, especially in women. For patients who do not tolerate RNAs, ACE2 inhibitors should be administered. If patients need potassium supplements, these will be replaced with MRA. It should be taken into account that in some patients, treatment with beta-blockers, nitrates, or PDE-5 does not have a favorable effect on increasing exercise capacity. If the patient remains symptomatic and is being treated with any of these drugs, discontinuation should be considered [6].

## 6. Future Therapeutic Directions

Numerous studies have clarified various pathophysiological aspects of ***HFpEF*** that contribute to the discovery of new therapeutic targets.

(a) metabolic therapy
(b) micro ARN.

### 6.1. Metabolic Therapy

**Melatonin** (also known as 5-Methoxy-N-acetyltryptamine) represents an indoleamine-derived molecule that is synthesized in the pineal gland, especially during night, and regulated by the hypothalamic suprachiasmatic nucleus. While the classical function of melatonin was mostly seen as that of a circadian rhythm, sleep-wake and body temperature cycles modulator, it also exhibits various other biological functions, such as anti-inflammatory, anti-aging, antioxidant, immunomodulatory, anti-excitatory, metabolic, and vasomotor activities [34]. Furthermore, melatonin exerts its physiological effects not only via a direct mechanism, using a receptor-dependent signaling pathway, but also indirectly, acting as a natural antioxidant that can reduce both reactive nitrogen and oxygen species by acting as a scavenger. The receptors for melatonin are G-protein coupled receptors, such as membrane receptors type 1 (MT1, Mel1A, MTNR1A) and type 2 (MT2, Mel1B, MTNR1B), as well as the retinoid-related orphan nuclear receptors RZR and RORa, with numerous signaling pathways being shown to mediate it’s downstream effects, such as adenylate cyclase, protein kinase C (PKC), phospholipase A2, guanylate cyclase, calcium channels, potassium channels, and phospholipase C [35].

The role of endogenous melatonin in the cardiovascular system is well-established at this point. Several papers found substantial decreases both in the synthesis and serum levels of melatonin in subjects suffering from various cardiovascular diseases, such as hypertension [36], coronary artery disease [37], and HF [38]. Melatonin also appears to play an important role in the regulation of nocturnal blood pressure [39] and can act as a protective agent in vascular disease by decreasing the expression of platelets and several adhesion molecules (CD31, ICAM-1, VCAM-1) while increasing nitric oxide (eNOS) [40]. Additionally, it improves the vasodilator function of small pulmonary arteries in patients with PH by enhancing the bioavailability and the vasodilator pathways associated with eNOS [41]. Likewise, melatonin supplementation has demonstrated a protective cardiovascular effect in experimental models of myocardial infarction [42], myocardial ischemia/reperfusion injury [43], left ventricular hypertrophy [44], and drug-induced cardiomyopathy [45].

Taking into consideration the lack of scientific evidence that supports efficient medical therapies for HFpEF and the necessity for a cost-effective and safe therapy, exogenous melatonin has been studied with increasing interest as a potential candidate for this purpose. Hence, it has been shown that melatonin levels negatively correlate with the levels of the N-terminal pro-brain natriuretic peptide (NT-pro-BNP) [46] and were, in consequence, studied in patients suffering from HFrEF [47]. Unfortunately, the success melatonin has shown in previous studies targeting cardiovascular diseases cannot be easily replicated for HFpEF, mainly due to a lack of efficiency in various distinct etiologies. Based on recent studies and given the heterogeneity of the syndrome, HFpEF is now understood not solely as a disease of the heart but also as a multiorgan chronic pathology, closely related to the obesity epidemic, the degenerative processes resulting from aging and the maladaptive mechanisms that result from different widespread pathologies, such as metabolic syndrome and diabetes mellitus [48]. The most well-understood biological effects that melatonin has been shown to possess and that can also impact the intricate pathophysiology of HFpEF are its antioxidative properties and its role in myocardial remodeling.

Oxidative stress occurs as a result of cellular redox imbalance, i.e., increased reactive oxygen species (ROS) production and/or decreased antioxidant availability. It can cause cardiomyocyte dysfunction by modifying proteins involved in excitation-contraction coupling and myocyte contraction, inducing various hypertrophy signaling kinases, mediating apoptosis, activating cardiac fibroblast proliferation, and activating the matrix metalloproteinases, thus causing ECM remodeling [49]. That given, melatonin and its metabolites demonstrated in previous studies a potent antioxidative effect, acting as a type I, type II, and type IV antioxidant [35]. Additionally, melatonin exerts its antioxidant effects via a cell surface receptor-independent pathway since the MT3 receptor is a QR2 (quinone oxidoreductase 2) cytosolic enzyme, reducing the generation of new free radicals, in particular reactive nitrogen and oxygen species. Thus, melatonin binding directly to the cytosolic QR2 catalytic site regulates its function by detoxifying and reducing the production of ROS Field [50], maintaining redox homeostasis, and protecting cells against molecular damage and oxidative stress [51]. Another mechanism by which oxidative stress has been shown to damage the heart is by interfacing with mitochondrial homeostasis [52]. In a study by Mingge Ding. et al., melatonin demonstrated antioxidant properties in diabetic hearts by inhibiting dynamin-related protein 1 (Drp1)-mediated mitochondrial fission [53]. Also, short-term melatonin supplementation was shown to reduce oxidative stress in myxomatous mitral valve degeneration in dogs but with no effect on left ventricular structure and function [54]. By the same token, Yuan Liu et al. demonstrated melatonin’s cardioprotective effect in HFpEF by attenuating obesity-induced myocardial oxidative stress and apoptosis and promoting the secretion of C1q/tumor necrosis factor-related protein 3 (CTRP3) by adipose tissue, thus ameliorating diastolic dysfunction in obesity-induced HFpEF [55].

Further, myocardial fibrosis is triggered by myocyte necrosis and inflammation and is also associated with LV hypertrophy. The latent association between melatonin and fibrosis was postulated by the fact that fibrosis can be detected in pinealectomized rats [56]. Melatonin is involved in the favorable evolution of fibrosis as a result of pathologies such as PH, myocardial infarction, and hypertension [57]. Hence, the association between melatonin treatment and the extent of fibrosis present in HF was shown in rats with isoproterenol-induced HF, in which the treatment with melatonin for 28 days resulted in a decreased level of oxidative stress, insoluble and total collagen, and the partial prevention of the beta-tubulin alteration in the LV, with the final results being a reduction in mortality and a prolonged average survival time [58]. Left ventricular hypertrophy (LVH) can also be partially prevented by melatonin in continuous light and L-NAME-induced left ventricular remodeling [59], but the results seem to be controversial since there are studies where it failed to reduce LVH, only affecting the myocardial fibrosis [60].

The gap in scientific data and the fact that the validation of the beneficial effects demonstrated on animal models is very difficult to obtain in human populations remain the main limiting factors in proposing melatonin as a suitable long-term treatment for HFpEF. Another major limiting factor is the lack of consensus regarding the specific details of melatonin treatment in terms of dosage, mode/route of administration, and the necessary expected period for the onset of the desired effects. Before we can propose melatonin as a suitable long-term treatment for HFpEF, future new studies are mandatory to have a better perspective. Melatonin appears to also play an important role in the regulation of nocturnal blood pressure.

**Serpina3**, also called α1-antichymotrypsin, is a protein belonging to the α1-globulin fraction, part of the family of protease inhibitors, with its gene expression regulated primarily by IL1 and IL6 cytokines via the STAT3 (signal transducer and activator of transcription 3) pathway. This protein is mainly produced by the liver and secreted into the circulation, but it can also be present in reduced concentration in the prostate, the testes, the pancreas, the gallbladder, the uterus, and the brain. Serpina3 acts primarily as a serine protease inhibitor, such as cathepsin G, chymotrypsins, and elastase, by binding them in a stable complex and protecting them from proteolytic activity [61].

Serpina3 has increasingly been studied for its role in the pathophysiology of HF, with conflicting opinions on the benefit/injury ratio of this protein in the deterioration of cardiac function. In a paper by Yuqing Tian et al. Serpina3 gene was found to be one of the six differentially expressed genes (DEG) related to HF [62], results that correlate with the paper by Jing Cao et al., which studied DEG present in ischemic cardiomyopathy [63]. Serpina3 was also postulated to play an important role in the relationship between epicardial adipose tissue (EAT) and HF. In pathological conditions, EAT may experience “phenotypic” transformations. Therefore, the increased volume and inflammation of EAT are associated with the progression of cardiac dysfunction in obese patients [64].

High blood concentration of α1-antichymotrypsin (>316 μg/mL), possibly secreted by the endothelial cells, was found to be associated with increased mortality or unplanned cardiac readmission in patients with de novo or worsened HF, regardless of EF [65]. Increased concentration of Serpina 3 also showed in previous studies an inhibitor effect on the accumulation of neutrophils in ischemic and reperfused myocardium and also helped to inactivate the cytotoxic metabolites released from neutrophils [65]. The hypothesis of the positive role of α1-antichymotrypsin in cardiovascular pathologies has also been strengthened based on the effect of LEX-032, a recombinant construct of human alpha 1-antichymotrypsin in which six amino acid residues were replaced with those of human alpha 1-protease inhibitor around the active center, which showed some beneficial effects in myocardial reperfusion injury in animal models by the inhibition of PMN-mediated cellular injury [66].

Cancer, next to stroke, is the second most important predictor of mortality in HFpEF, with a 2.5-fold increased mortality risk [67]. In a recent study, Wouter C Meijers et al. demonstrated a causal relationship between HF and tumor growth and found Serpina3 to be the most promising culprit. Serpina3 stimulates the proliferation of colon tumor cells in vitro via an Akt (also known as protein kinase B)-dependent pathway, a signal transduction pathway that promotes survival and growth in response to extracellular signals [68].

The fact that Serpina3 plays a role in the pathophysiology of HF is undeniable. At the moment, there is no consensus on the role that Serpina3 will play in the long-term treatment of HFpEF, as a beneficial treatment based on its immunomodulatory properties, or as a potential target to reduce long-term cancer mortality.

### 6.2. Genetic Therapies

So far, no therapy backed up by scientific evidence has proved to increase survival in HFpEF, the treatment goals being an increase in functional status and a reduction in symptomatology and risk of re-hospitalization [69,70]. However, in the last decade, many scientists have focused on the early diagnostic and therapeutic management of HFpEF. Unfortunately, it is still portrayed as a condition with a very negative prognosis. Therefore, efforts have shifted towards defining specific phenotypes within the HFpEF and developing targeted treatment approaches via cardiac gene therapy that could represent a great alternative to current treatment strategies. Scientists proposed a method for the distribution of cell-directed transgenes (exogenous genes) that would eventually generate “good” proteins capable of counteracting the downregulations implicated in the pathophysiology of HF [71]. In the subsequent section, we will present various structures that could be targeted or used in HFpEF genetic therapy.

The sarcoplasmic/endoplasmic reticulum Ca^2+^ (SERCA) plays a crucial role in the myocardial cell function as it regulates the release and re-uptake of calcium ions (Ca^2+^) and, in consequence, the contraction/relaxation of the heart muscle. The activity and expression of SERCA-ATPase-2a (SERCA2a), a subtype of protein pump expressed in the cardiac myocytes, appears to be decreased in HF, leading to impaired relaxation and predisposition to arrhythmias, major pathological “hallmarks” of HFpEF. Considering this, an augmented expression of SERCA2a via gene therapy has been proven to be effective in isolated human cardiomyocytes and experimental models [71,72]. The vector chosen to carry SERCA2a transgenes was represented by the adeno-associated virus of serotype 1 (AAV1). However, the procedure did not meet the expected results, as it had no significant effect on clinical (NYHA functional class, 6-min walk test distance) or laboratory endpoints (NT-proBNP levels). In consequence, the first clinical trial regarding gene therapy in HF failed when reaching final phase 2b (CUPID-Calcium Up-Regulation by Percutaneous Administration of Gene Therapy in Cardiac Disease) [71]. Furthermore, proteins involved in calcium homeostasis are regulated by micro-RNAs (small, single-stranded, non-coding RNA molecules), which appear to be downregulated in failing hearts. For example, a reduction in microRNA-1 was described in heart conditions such as dilated and ischemic cardiomyopathy. Also, studies revealed that the delivery of microRNA-1 via AAV9 in rats with induced hypertrophy enhances contractile function by restoring intracellular Ca^2+^. Consequently, intense cardiac remodeling was also revealed by the up-regulation of other micro-RNAs (microRNA-24, microRNA-125b, microRNA-195, microRNA-199a, and microRNA-214), in both patients with end-stage HF and experimental animals [73].

MicroRNAs represent a form of non-coding RNAs with very stable sequences that regulate gene expression (through the inhibition of messenger RNA transcription or the deterioration of targeting proteins). Studies revealed that during hypoxia, microRNA-210 was significantly upregulated, playing a protective role in cardiac remodeling by inhibiting apoptosis and stimulating cell proliferation, migration, differentiation, and angiogenesis. It is easy to understand why microRNA-210 expression is altered in HF, and its overexpression would lead to cardio-protective effects. Its overexpression is triggered by hypoxia-inducible factors (HIF), such as HIF-1α, found in most cell lines. It was also reported that microRNA-210 levels in mononuclear cells from patients with HF are also associated with NT-proBNP, a hallmark molecule of HF conglomerate. Even though no significant correlation was found between NT-proBNP and microRNA-210 (in NYHA-2 patients’ plasma), the microRNA-210 level seemed to be decreased in patients with improved NT-proBNP [74]. A meta-analysis consisting of 45 studies evaluated the diagnostic efficiency of microRNAs in HF, revealed that it does not possess any superiority compared to regular biomarkers, but reinforced the observation that in HFpEF, it can improve the diagnostic power of NT-proBNP, which has a very low sensitivity in this condition [75].

Moreover, it appears that there is a trend of increased levels of microRNA-210 in the long-living individuals (defined as patients over 90 years old), as compared to younger individuals, but interestingly, the upregulation was found in the non-healthy nonagenarian group. Looking forward to genetic therapy perspectives, experimental models have successfully identified various drugs that can upregulate microRNA-210. One promising substance is crocin (extracted from saffron, a plant long used in traditional medicine that possesses antioxidant, anti-inflammatory, antiatherosclerotic, and anticancer properties. For instance, the administration of a single dose of crocin in rats, especially when combined with voluntary exercise for eight weeks, was followed by a significant increase in microRNA-210 in the cardiac tissue, underlying the potential of crocin as a new therapeutic agent that should not be underrated [74]. The cardioprotective effects of crocin were also confirmed by other scientists in patients treated with doxorubicin (a chemotherapeutic drug) [76], and also by other experimental studies that proved its ability to regulate heart angiogenesis via the upregulation of not only microRNA-210 but also microRNA-126 [77]. In addition, ventricular dysfunctions appear to be prevented by the inhibition of another microRNA, named microRNA-146, which leads to a decrease in apoptosis at the myocyte level. It was also shown to regulate endothelial angiogenesis and heart regeneration, proving its importance as a target for future therapies [78].

One potential therapeutic target could be the stromal cell-derived factor 1 (SDF-1), which induces cardiac repair via intrinsic repair processes, even though it has no direct action on cardiac function [71]. On the opposite pole, Philippaert et al. suggested that the cardioprotective effect of SGLT2 inhibitors relies on a molecular mechanism. They demonstrated that the beneficial effects on cardiac function are preserved even in patients without diabetes. Therefore, the effects must be independent of the improvement in glycemic levels. The mechanism was put on display in HF nondiabetic mice, which is also associated with the inhibition of the cardiac nuclear-binding domain-like receptor 3 (NLRP3) inflammasome, making it a possible target for gene therapy [79].

Adenylyl cyclase (AC6) represents an enzyme that transforms ATP (adenosine triphosphate) into cyclic adenosine monophosphate (cAMP), which is fundamental to cardiac function via the modulation excitation-contraction coupling and inotropic action. Experimental gene transfer of AC6 and its overexpression in animals with HF led to a reversal of pathological LV remodeling, improved cardiac function, and even a reduction in arrhythmic events [71].

In addition, the impairment of soluble guanylate-cyclase (sGC) in HF, the enzyme responsible for cyclic guanosine-monophosphate (cGMP) formation, raised interest among scientists who sought to evaluate its power as a new therapeutic target. Filippatos G and colleagues revealed that higher doses of vericiguat, a novel oral sGC stimulator, strongly improved patients’ functional status (via patients’ reported outcomes) but had no significant effect on the chosen primary end-points such as NT-proBNP or left atrial volume [80].

Another oral sGC stimulator, riociguat, showed significant favorable hemodynamic effects in patients with HFpEF and PH, previously unexposed to vasoactive treatment. This last scientific paper reported that even though there was a significant improvement in cardiac output and pulmonary vascular resistance, the favorable hemodynamic effects were not accompanied by significant improvements in NT-proBNP or clinical status within the study period [81] (Table 1).

Moving forward, other RNA molecules derived from pre-microRNAs, known as circular RNAs (circARNs), have been extensively studied due to their implications in heart diseases [82]. They present a shape very much like a circle and proved to be valuable potential biomarkers and therapeutic targets given their stable nature [82]. In terms of future genetic therapies, synthesized circRNAs could offer superior controllability as compared to endogenous circRNAs. Through the artificial incorporation of microRNAs into the circRNAs sequence, scientists have successfully obtained circRNAs that target particular microRNAs, thus providing a therapeutic approach with important clinical applications in the progression of HF and other cardiovascular diseases [83].

Recently, there was a broad focus on epicardial adipose tissue (EAT) due to its relationship with chronic systemic inflammation, which is believed to play a key role in the pathogenesis of HFpEF. It is well established that adipose cells, through their secretory function, can lead to microvascular dysfunction, fibrosis, and, thus, cardiac remodeling. Correspondingly, anti-inflammatory and antihyperglycemic medication proved to have beneficial effects on EAT in patients with HFpEF and atrial fibrillation but not in patients with HFrEF [84]. Recent studies revealed that EAT can secrete, besides cytokines, exosomes that carry non-coding RNAs. He et al. [85] investigated the expression pattern of circRNAs in the EAT of HFpEF subjects. The study identified 131 circRNAs by RNA-sequencing method and showed that their up-regulation is mainly related to various cellular processes such as metabolic, macromolecule biosynthesis, protein binding response, transferase activity, and catalytic. Interestingly, one particular circRNA (Hsa_circ_0005583) proved to originate from the Ataxia-Telangiesctasia Mutated (ATM) gene locus on chromosome 11, which helps regulate the cell cycle and repairs damaged DNA [85]. ATM gene was shown to also decrease life expectancy in oncologic patients with heart disease. Hence, it is of great importance for scientists to clearly define in future studies the role of Hsa_circ_0005583 and its relationship with ATM.

Although the degree of involvement of circRNA-protein binding in heart development has not yet been established, it appears to play a significant role in the regulatory pathways of various cells implicated in cardiovascular diseases. In atherosclerosis, microRNA binding directly to human RNA-binding protein (HuR) regulates the formation of IL-6, TNF-α, IL-1β, and myocardial fibrosis via fibroblast activation and expansion [86]. Thus, targeting proteins dedicated to HuR binding and inhibiting their role in the inflammation process could lead to a reduction in the progression of atherosclerosis. Likewise, it was stipulated that other proteins (such as lncRNA CCRR) binding to CIP85 (Cx43- interacting protein of 85-kDa) influence cardiac myocyte function and predispose to cardiac electrical anomalies and arrhythmias in HF patients, but the precise mechanism remains unclear [86].

A study evaluating microRNA’s expression in the left ventricular tissue, obtained from HFpEF patients subjected to open heart surgery, identified 152 significantly differentially expressed microRNAs compared to control patients (*p* < 0.05). Various mechanisms contributing to HF development were linked to the downregulation of hsa-miR-848 and upregulation of hsa-miR-21-3p and hsa-miR-193b [87].

Jankauskas SS et al. observed that microRNA-181c was significantly upregulated (*p* < 0.0001) in HFpEF patients vs. controls and confirmed via studies in vitro that it is targeting encoding Parkin (PRKN), and “Mothers Against Decapentaplegic Homolog 7 (SMAD7) in human cardiac fibroblasts, genes involved in the ubiquitin-proteasome system and cell growth processes [88].

Ghosh et al. proved in experimental models of non-ischemic diabetic hearts that the therapeutic breakdown of microRNA-320, which acts as an apoptosis promoter, partially restored the deteriorated cardiac function [89].

Similarly, the breakdown of microRNA-200c-3p in experimental animals subjected to chronic cardiac pressure overload resulted in a partially reversed process of wall hypertrophy, less fibrotic areas, and preserved cardiac EF [90].

Another important finding of Shao S et al. may have highlighted the mechanism by which ivabradine acts as a cardioprotective agent. It appears that ivabradine upregulates microRNA-133a, which is implicated in regulating connective tissue growth factor and collagen 1 in cardiac myofibroblasts [91].

This research on microRNA, which inhibits the induction of inflammation, microvascular dysfunction, and myocardium fibrosis, has been performed. Unfortunately, it has not shown definite effectiveness up to now.

**Table 1:** Novel emerging therapies for HFpEF.

No.	Genetic Targets	Expression	Function	Study
1.	SERCA-ATPase-2a (SERCA2a)	Up-regulation	Expression of SERCA2a in isolated human cardiomyocytes and experimental models	Gabisonia K et al. [71] Zhihao L et al. [72]
			No significant effects on clinical (NYHA functional class, 6-min walk test distance) or laboratory endpoints (NT-pro-BNP levels)	Gabisonia K et al. [71]
2.	MicroRNAs		Great capacity for ‘ruling out’ patients with HFrEF or HFpEF	Parvan R et al. [75] (meta-analysis consisting of 45 studies)
3.	MicroRNA-1	Up-regulation via AAV9	Enhances contractile function by restoring intracellular Ca^2+^	Park JH et colleagues. [73]
4.	MicroRNA-24 MicroRNA-125b MicroRNA-195 MicroRNA-199a	Up-regulation	Intense cardiac remodeling in patients with end-stage HF and experimental models	
5.	MicroRNA-210		Protective role in cardiac remodeling by inhibiting apoptosis and stimulating cell proliferation, migration, differentiation, and angiogenesis.	Guan Y et al. [74]
		Down-regulation	Altered expression in HF	
		Down-regulation	Decreased levels in patients with improved NT-pro-BNP	
		Up-regulation	Increased levels in the long living individuals (>90 years old)	
			Significantly increased by crocin (antioxidant, antiinflammatory, antiatherosclerotic)	Guan Y et al. [74] Razmaraii N et al. [75] Ghorbanzadeh V et al. [77]
6.	MicroRNA-146	Inhibition	Prevents ventricular dysfunctions	Mahdavi FS et al. [78]
			Decreases apoptosis at the myocyte level	
			Regulates endothelial angiogenesis and heart regeneration	
7.	CircARNs	Up-regulation	In the EAT of HFpEF subjects	He et al. [85]
			In cellular processes such as metabolic, macromolecule biosynthesis, protein binding response, transferase activity and catalytic	
			Helps regulating cell cycle and repairs damaged DNA	
			Regulates the formation of IL-6, TNF-α, IL-1β via HuR	Zhao H et colleagues. [86]
			Regulates myocardial fibrosis via fibroblasts activation and expansion via Hur	
8.	SDF-1		Induces cardiac repair	Gabisonia K et al. [71]
9.	NLRP3	Inhibition	Beneficial effects on cardiac function via SGLT2 inhibitors	Philippaert K et al. [79]
10.	AC6	Up-regulation	Reversal of pathological LV remodeling	Gabisonia K et al. [71]
			Reduction in arrhythmic events	
11.	sGC	Stimulation	Generates cGMP in HF	Filippatos G et al. [12] Dachs TM et al. [13]
			Strongly improved patient’s functional status via vericiguat	
			No significant effect on NT-pro-BNP or left atrial volume	
			Significant improvement in cardiac output and pulmonary vascular resistance in patients with HFpEF via riociguat	

AC6: Adenylyl cyclase 6, cGMP: cyclic guanosine-monophosphate, MicroRNAs, CircARNs: circular ribonucleic acids, HFrEF: Heart failure with reduced ejection fraction, HFpEF: Heart failure with preserved ejection fraction, HuR: human RNA-binding protein, IL-1β: interleukin 1 beta, IL-6: interleukin6, LV: Left Ventricle, NLRP3—cardiac nuclear-binding domain-like receptor 3, NT-pro-BNP: N terminal pro-natruretic brain peptide, NYHA: New York Heart Association, EAT: epicardial adipose tissue, SDF-1—stromal cell-derived factor 1, SERCA2a: Sarcoplasmic/endoplasmic reticulum Ca^2+^, SGLT2: Sodium-Glucose Transport Protein 2, sGC: soluble guanylate-cyclase, TNF-α; Tumor Necrosis Factor alpha.

## 7. Conclusions

Therapies with proven efficacy in patients with HFpEF are diuretics, SGLT2 inhibitors, and the treatment of comorbidities. Numerous studies have clarified the various pathophysiological aspects of HFpEF. There are potential drugs or therapies that can be used in the future: a soluble guanylate cyclase stimulator, agents that loosen the bonds between proteins and glycosylation end products, and gene therapy to increase the level of SERCA2 protein in the myocardium. As researchers, it is our duty to ask more questions, explore the mechanisms at play in HFpEF, and consider the clinical implications. Only through rigorous investigation and collaboration can we hope to unravel the mysteries surrounding HFpEF. The uncertainties we face today are the opportunities for breakthroughs tomorrow.

## Data Availability

Not applicable.

## Abbreviations

AAVS1: Adeno-associated virus of serotype 1, AC6: Adenylyl cyclase 6, cAMP: cyclic adenosine- monophosphate, cGMP: cyclic guanosine-monophosphate, CircARNs: circular ribonucleic acids, DEG: differentially expressed genes, HFrEF: Heart failure with reduced ejection fraction, HFpEF: Heart failure with preserved ejection fraction, HuR: human RNA-binding protein, IL-1β: interleukin 1 beta, IL-6: interleukin6, LV: Left Ventricle, MRA: mineralocorticoid receptor antagonist, NLRP3—cardiac nuclear-binding domain-like receptor 3, NT-pro-BNP: N terminal pro-natruretic brain peptide, NYHA: New York Heart Association, EAT: epicardial adipose tissue, PDE-5: phosphodiesterase 5, SDF-1—stromal cell-derived factor 1, SERCA2a: Sarcoplasmic/endoplasmic reticulum Ca^2+^, SGLT2: Sodium-Glucose Transport Protein 2, sGC: soluble guanylate-cyclase, TNF-α; Tumor Necrosis Factor alpha.
